# The essential glucose transporter GLUT1 is epigenetically upregulated by C/EBPβ and WT1 during decidualization of the endometrium

**DOI:** 10.1016/j.jbc.2021.101150

**Published:** 2021-08-31

**Authors:** Isao Tamura, Taishi Fujimura, Yumiko Doi-Tanaka, Haruka Takagi, Yuichiro Shirafuta, Takuya Kajimura, Yumiko Mihara, Ryo Maekawa, Toshiaki Taketani, Shun Sato, Hiroshi Tamura, Norihiro Sugino

**Affiliations:** Department of Obstetrics and Gynecology, Yamaguchi University Graduate School of Medicine, Ube, Japan

**Keywords:** glucose transport, glucose transporter 1 (GLUT1), CCAAT/enhancer-binding protein β (C/EBPβ), Wilms’ tumor 1(WT1), histone acetylation, endometrial stromal cell, decidualization, epigenetics, E1A binding protein p300 (p300), C/EBPβ, CCAAT enhancer-binding protein β, ESC, endometrial stromal cell, FBS, fetal bovine serum, FOXO1, forkhead box O1, GLUT1, glucose transporter 1, HAT, histone acetyltransferase, IGFBP-1, IGF-binding protein-1, PRL, prolactin, VLDLR, very low-density lipoprotein receptor, WT1, Wilms’ tumor 1

## Abstract

Human endometrial stromal cells (ESCs) differentiate into decidual cells by the action of progesterone, which is essential for implantation and maintenance of pregnancy. We previously reported that glucose uptake by human ESCs increases during decidualization and that glucose is indispensable for decidualization. Although glucose transporter 1 (GLUT1) is upregulated during decidualization, it remains unclear whether it is involved in glucose uptake. Here, we attempted to determine the role of GLUT1 during decidualization as well as the factors underlying its upregulation. ESCs were incubated with cAMP to induce decidualization. Knockdown of GLUT1 suppressed cAMP-increased glucose uptake and the expressions of specific markers of decidualization, IGF-binding protein-1 (IGFBP-1), and prolactin (PRL). To investigate the regulation of GLUT1 expression, we focused on CCAAT enhancer-binding protein β (C/EBPβ) and Wilms’ tumor 1 (WT1) as the upstream transcription factors regulating GLUT1 expression. Knockdown of either C/EBPβ or WT1 suppressed cAMP-increased GLUT1 expression and glucose uptake. cAMP treatment also increased the recruitment of C/EBPβ and WT1 to the GLUT1 promoter region. Interestingly, cAMP increased the H3K27 acetylation (H3K27ac) and p300 recruitment in the GLUT1 promoter region. Knockdown of C/EBPβ or WT1 inhibited these events, indicating that both C/EBPβ and WT1 contribute to the increase of H3K27ac by recruiting p300 to the GLUT1 promoter region during decidualization. These findings indicate that GLUT1 is involved in glucose uptake in ESCs during decidualization, thus facilitating the establishment of pregnancy.

Human endometrial stromal cells (ESCs) undergo cyclic changes during the menstrual cycle in response to changing levels of steroid hormones. Decidualization is one of these changes in which ESCs differentiate into decidual cells by the action of progesterone, which is essential for implantation and maintenance of pregnancy (1, 2, 3). Our previous genome-wide analyses revealed that a number of genes are up- or downregulated by decidualization in human ESCs (4, 5). These changes are accompanied by dramatic metabolic changes including increases in the storage of lipids (6) and glycogen (7).

We previously reported that glucose uptake increases during decidualization and that glucose is indispensable for decidualization (5, 8). The efficiency of glucose uptake is determined by a family of facilitative glucose transporters (GLUTs) (9). Among several GLUTs identified in the human endometrium, GLUT1 is highly expressed in ESCs, and its expression increases during decidualization (10, 11). Although these findings suggest that GLUT1 is involved in the glucose uptake of ESCs, none of previous reports have proved it. In addition, the mechanisms by which GLUT1 is upregulated during decidualization have not been well clarified. Although various transcription factors are activated to upregulate many genes during decidualization (4, 12, 13, 14, 15), it remains unclear which transcription factors are involved in the regulation of GLUT1 expression. By performing a transcriptome analysis, we previously revealed that CCAAT enhancer-binding protein β (C/EBPβ) and Wilms’ tumor 1(WT1) are important transcription factors regulating a number of decidualization-related genes (4, 6), which led us to focus on these transcription factors as upstream regulators of GLUT1 during decidualization.

Gene expression including transcription involves a change of chromatin structure, which can be regulated by epigenetic mechanisms such as histone modifications (16, 17, 18). Acetylation of histone-H3 lysine-27 (H3K27ac) is one of the histone modifications that activates gene transcription and is highly enriched in the active promoter or enhancer regions (19). We found that decidualization stimulus upregulates a number of gene expressions with the genome-wide increase of H3K27ac levels (4, 5, 20). Interestingly, these H3K27ac-regulated genes were enriched in the insulin signaling pathway (5). We also reported that glucose contributes to increasing the H3K27ac levels of the promoter regions of IGF-binding protein-1 (IGFBP-1), prolactin (PRL), and forkhead box O1 (FOXO1), which are well-recognized markers for decidualization (8). These findings suggested that H3K27ac is associated with glucose metabolisms. Furthermore, our recent ChIP-sequence analysis indicated that the H3K27ac level of the GLUT1 promoter region increases during decidualization (4). Therefore, we hypothesized that the change of H3K27ac is an important mechanism for the regulation of GLUT1 and the following glucose uptake during decidualization.

Here, we show that GLUT1 is involved in the increase of glucose uptake during decidualization. We also found that C/EBPβ and WT1 are important transcription factors regulating GLUT1 expression during decidualization and that GLUT1 expression is epigenetically regulated.

## Results

### GLUT1 expression in human endometrium and ESCs, and how it is affected by cAMP

**Figure 1:**
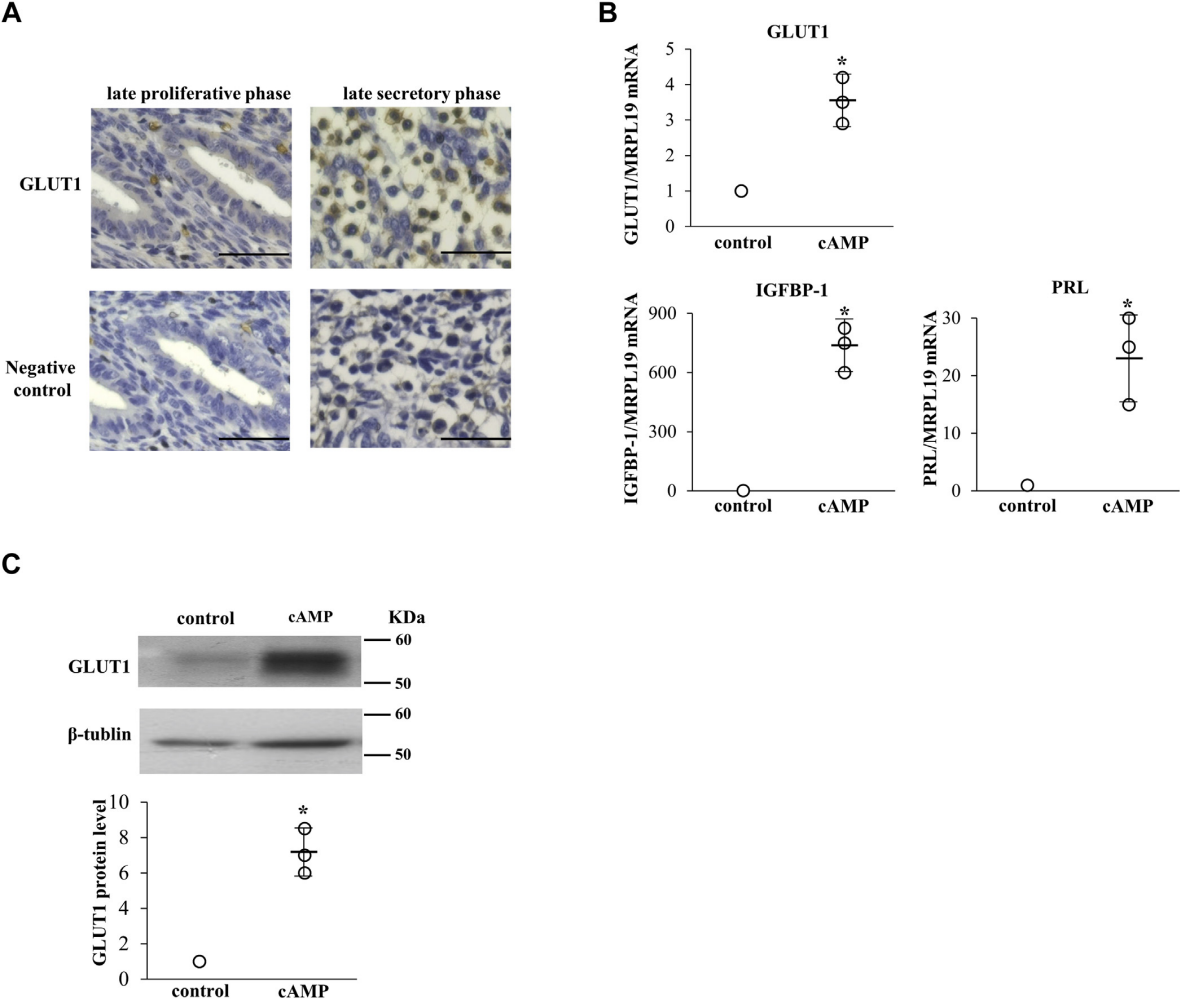
**GLUT1 expression in human endometrium and ESCs, and how it is affected by cAMP.***A*, immunohistochemical expression of GLUT1 in the late proliferative and secretory phase endometrium. The photographs in the lower row are negative controls in each sample. Scale bars, 50 μm. *B*, ESCs were treated with or without cAMP (0.5 mM) for 4 days. Cells treated without cAMP were used as the control. mRNA expression levels were analyzed by real-time RT-PCR. Values of GLUT1, IGFBP-1, PRL were normalized to those of MRPL19 and expressed as a ratio of the control sample. Data are mean ± SD of three different incubations. ∗*p* < 0.01 *versus* control (unpaired *t* test). *C*, whole-cell lysates were prepared and subjected to Western blotting to examine GLUT1 protein expression. β-tubulin was used as an internal control. The immunoblot is a representative of three different incubations. Western blot bands were quantified by ImageJ and values of GLUT1 were normalized to those of β-tubulin and expressed as a ratio of the control sample. Data are mean ± SD of three different incubations. ∗*p* < 0.01 *versus* control (unpaired *t* test).

The expression of GLUT1 in the human endometrium was examined by immunohistochemistry (Fig. 1*A*). GLUT1 was expressed in stromal cells obtained from the late proliferative phase endometrium, but was expressed more strongly in the stromal cells that were morphologically identified as predecidual cells in the late secretory phase endometrium. The expression of GLUT1 in human ESCs was examined by real-time RT-PCR and Western blotting. cAMP increased the mRNA (Fig. 1*B*) and protein expression levels (Fig. 1*C*) of GLUT1 with the induction of IGFBP-1 and PRL mRNA in ESCs, which are specific markers of decidualization (Fig. 1*B*).

### Involvement of GLUT1 in glucose uptake during decidualization

**Figure 2:**
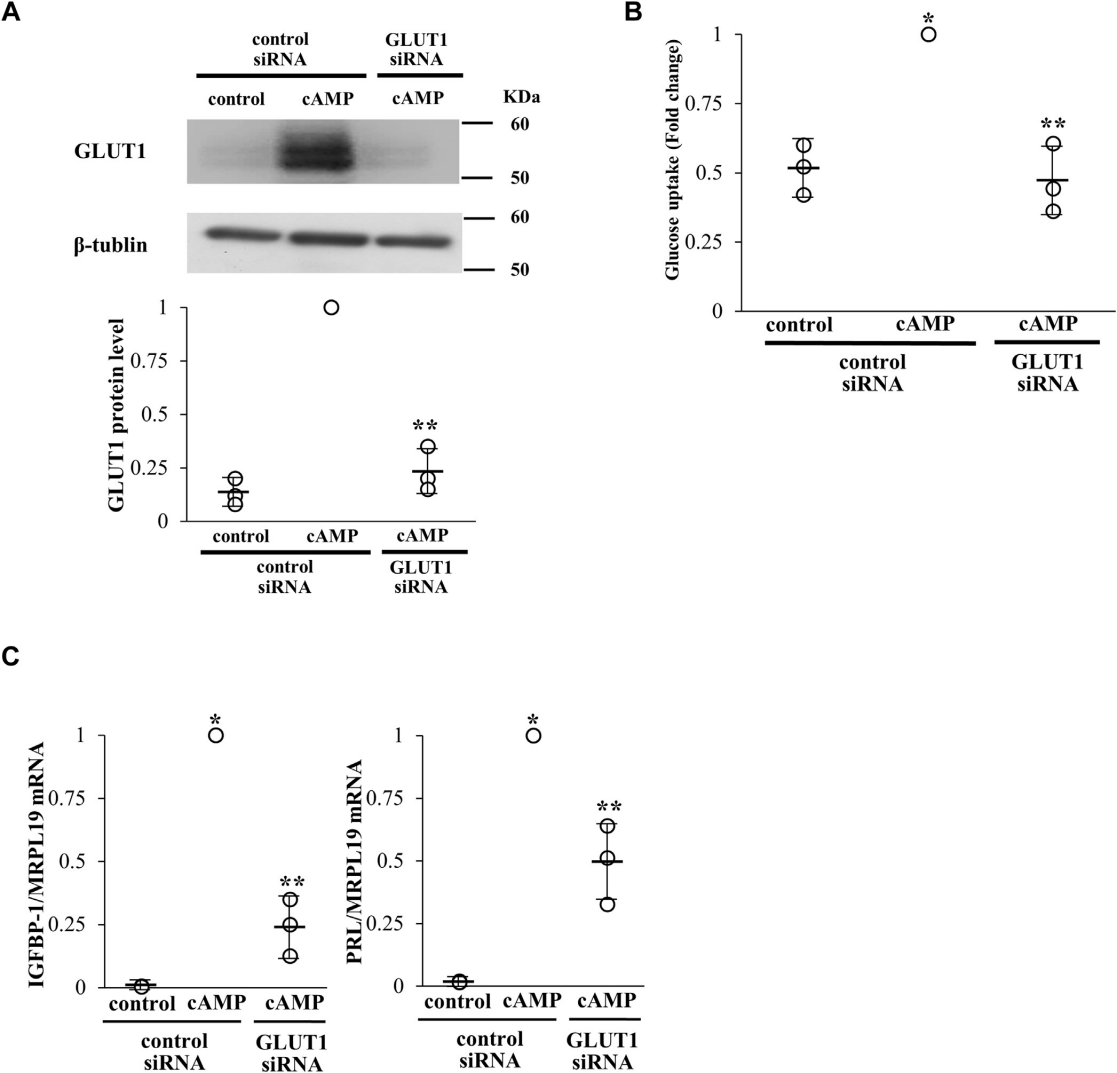
**Involvement of GLUT1 in glucose uptake in ESCs during decidualization.***A*, ESCs were transfected with a siRNA targeted against GLUT1 or with a nontargeting siRNA as a control. Forty-eight hours after siRNA transfection, ESCs were treated with or without cAMP for 4 days. Whole-cell lysates were prepared and subjected to Western blotting to examine the knockdown of GLUT1 protein expression. β-tubulin was used as an internal control. The immunoblot is a representative of three different incubations. Western blot bands were quantified by ImageJ and values of GLUT1 were normalized to those of β-tubulin and expressed as a ratio of the cAMP treatment sample in the control siRNA sample. Data are mean ± SD of three different incubations. ∗*p* < 0.01 *versus* control treatment in the control siRNA; ∗∗*p* < 0.01 *versus* cAMP treatment in the control siRNA (Tukey–Kramer test). *B*, effect of cAMP and GLUT1 knockdown on glucose uptake. The ability of glucose uptake was examined by a 2-DG uptake assay and expressed as a ratio of the cAMP treatment sample in the control siRNA sample. Data are mean ± SD of three different incubations. ∗*p* < 0.01 *versus* control treatment in the control siRNA; ∗∗*p* < 0.01 *versus* cAMP treatment in the control siRNA (Tukey-Kramer test). *C*, involvement of GLUT1 in cAMP-induced gene expressions of IGFBP-1 and PRL. mRNA expression levels were analyzed by real-time RT-PCR. Values of IGFBP-1 and PRL were normalized to those of MRPL19 and expressed as a ratio of the cAMP treatment sample in the control siRNA sample. Data are mean ± SD of three different incubations. ∗*p* < 0.01 *versus* control treatment in the control siRNA; ∗∗*p* < 0.01 *versus* cAMP treatment in the control siRNA (Tukey–Kramer test).

To examine whether the glucose uptake during decidualization is mediated by GLUT1, GLUT1 was knocked down by siRNA. cAMP-increased GLUT1 protein expression was suppressed by GLUT1 siRNA treatment (Fig. 2*A*). Glucose uptake was increased by cAMP, and this increase was abolished by GLUT1 knockdown (Fig. 2*B*). In addition, GLUT1 knockdown suppressed the cAMP-induced expressions of IGFBP-1 and PRL (Fig. 2*C*). Considering our previous finding that glucose is necessary for decidualization (5, 8), the present results show that GLUT1 contributes to decidualization by regulating glucose uptake in ESCs.

### Involvement of C/EBPβ and WT1 in the regulation of GLUT1 and glucose uptake during decidualization

**Figure 3:**
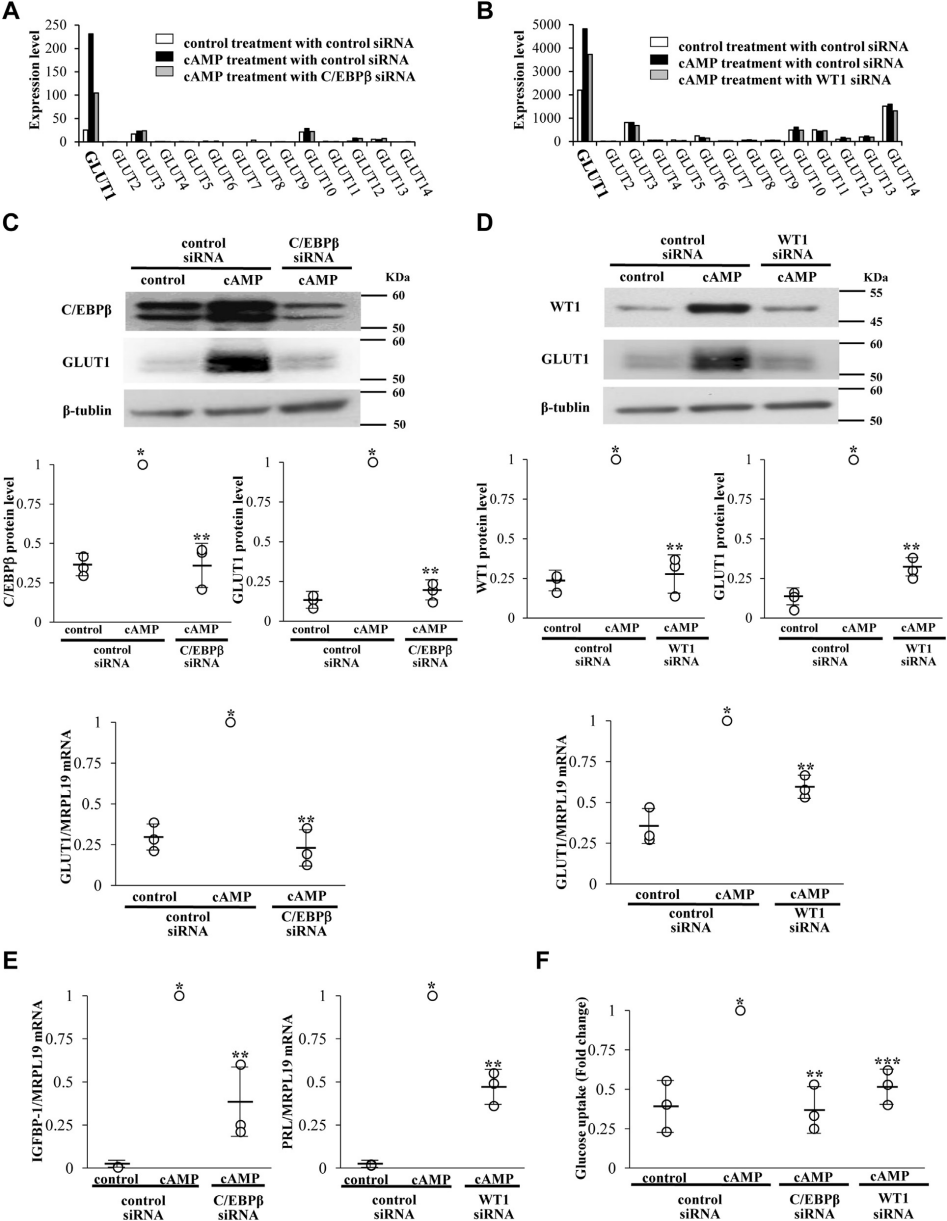
**Involvement of C/EBPβ and WT1 in the regulation of GLUT1 and glucose uptake during decidualization.***A*, expression levels of 14 GLUT family members. mRNA expression levels of GLUTs were extracted from previously published RNA-sequence data that examined the effect of cAMP and C/EBPβ knockdown in ESCs. Gene expression levels of each gene are expressed as Reads Per Kilobase of exon per Million mapped reads (RPKM). *B*, mRNA expression levels of GLUTs were extracted from previously published microarray data that examined the effect of cAMP and WT1 knockdown in ESCs. Gene expression levels of each gene are expressed as microarray signals. *C*, involvement of C/EBPβ in cAMP-increased gene expression of GLUT1. Forty-eight hours after siRNA transfection against C/EBPβ, ESCs were treated with or without cAMP for 4 days. Protein expression levels and mRNA expression levels were analyzed by Western blotting and real-time RT-PCR, respectively. The immunoblot is a representative of three different incubations (*upper panel*). Western blot bands were quantified by ImageJ and values of C/EBPβ and GLUT1 were normalized to those of β-tubulin and expressed as a ratio of the cAMP treatment sample in the control siRNA sample (*middle panel*). Values of GLUT1 mRNA were normalized to those of MRPL19 and expressed as a ratio of the cAMP treatment sample in the control siRNA sample (*lower panel*). Data are mean ± SD of three different incubations. ∗*p* < 0.01 *versus* control treatment in the control siRNA; ∗∗*p* < 0.01 *versus* cAMP treatment in the control siRNA (Tukey–Kramer test). *D*, involvement of WT1 in cAMP-increased gene expression of GLUT1. Forty-eight hours after siRNA transfection against WT1, ESCs were treated with or without cAMP for 4 days. Protein expression levels and mRNA expression levels were analyzed by Western blotting and real-time RT-PCR, respectively. The immunoblot is a representative of three different incubations (*upper panel*). Western blot bands were quantified by ImageJ and values of WT1 and GLUT1 were normalized to those of β-tubulin and expressed as a ratio of the cAMP treatment sample in the control siRNA sample (*middle panel*). Data are mean ± SD of three different incubations. ∗*p* < 0.01 *versus* control treatment in the control siRNA; ∗∗*p* < 0.01 *versus* cAMP treatment in the control siRNA (Tukey–Kramer test). *E*, involvement of C/EBPβ and WT1 in cAMP-induced gene expressions of IGFBP-1 and PRL. mRNA expression levels were analyzed by real-time RT-PCR. Values of IGFBP-1 and PRL were normalized to those of MRPL19 and expressed as a ratio of the cAMP treatment sample in the control siRNA sample. Data are mean ± SD of three different incubations. ∗*p* < 0.01 *versus* control treatment in the control siRNA; ∗∗*p* < 0.01 *versus* cAMP treatment in the control siRNA (Tukey–Kramer test). *F*, involvement of C/EBPβ and WT1 in glucose uptake during decidualization. Effect of cAMP and the knockdown of C/EBPβ or WT1 on glucose uptake were examined by a 2-DG uptake assay. The abilities of glucose uptake were expressed as a ratio of the cAMP treatment sample in the control siRNA sample. Data are mean ± SD of three different incubations. ∗*p* < 0.01 *versus* control treatment in the control siRNA; ∗∗*p* < 0.01 *versus* cAMP treatment in the control siRNA. ∗∗∗*p* < 0.05 *versus* cAMP treatment in the control siRNA (Tukey–Kramer test).

We previously performed two transcriptome analyses (an RNA-sequence analysis and a microarray analysis) to identify the genes regulated by C/EBPβ and WT1 in human ESCs during decidualization (4, 6). In both analyses, total RNAs were extracted from the three groups (no treatment with control siRNA, cAMP treatment with control siRNA, and cAMP treatment with C/EBPβ or WT1 siRNA). The GLUT family has 14 members (9). Their expression values were determined from the transcriptome data (Fig. 3). Figure 3*A* shows the effects of cAMP and C/EBPβ knockdown on the expression levels of GLUTs. GLUT1 was expressed in nondecidualized ESCs (control treatment with control siRNA) and was highly upregulated in decidualized ESCs (cAMP treatment with control siRNA). On the other hand, the expression levels of other GLUTs were very low or not detected in both nondecidualized and decidualized ESCs. C/EBPβ knockdown suppressed cAMP-increased GLUT1 expression (cAMP treatment with C/EBPβ siRNA). Similar results were observed in the transcriptome analysis examining the effect of WT1 knockdown (Fig. 3*B*). These results indicated that GLUT1 is the most important glucose transporter among GLUT members during decidualization of human ESCs, and its upregulation is mediated by C/EBPβ and WT1. The results of transcriptome analysis were validated by real-time RT-PCR and Western blotting. C/EBPβ knockdown completely suppressed the cAMP-increased GLUT1 mRNA and protein expressions, whereas WT1 knockdown partially suppressed them (Fig. 3, *C* and *D*), suggesting that other transcription factors than C/EBPβ and WT1 are also involved in cAMP-increased GLUT1 expressions and that their expressions or recruitments to GLUT1 promoter are regulated by C/EBPβ. Furthermore, knockdown of C/EBPβ or WT1 suppressed the cAMP-induced expressions of IGFPB1 and PRL (Fig. 3*E*), which is consistent with our previous reports showing the involvement of these transcription factors in decidualization (15, 21). We examined whether C/EBPβ and WT1 are involved in the regulation of glucose uptake during decidualization. cAMP-increased glucose uptake was significantly inhibited by the knockdown of C/EBPβ or WT1 (Fig. 3*F*). These results showed that C/EBPβ and WT1 are upstream regulators of GLUT1 and contribute to increasing glucose uptake during decidualization.

### Decidualization-induced recruitment of C/EBPβ and WT1 to the GLUT1 promoter region

**Figure 4:**
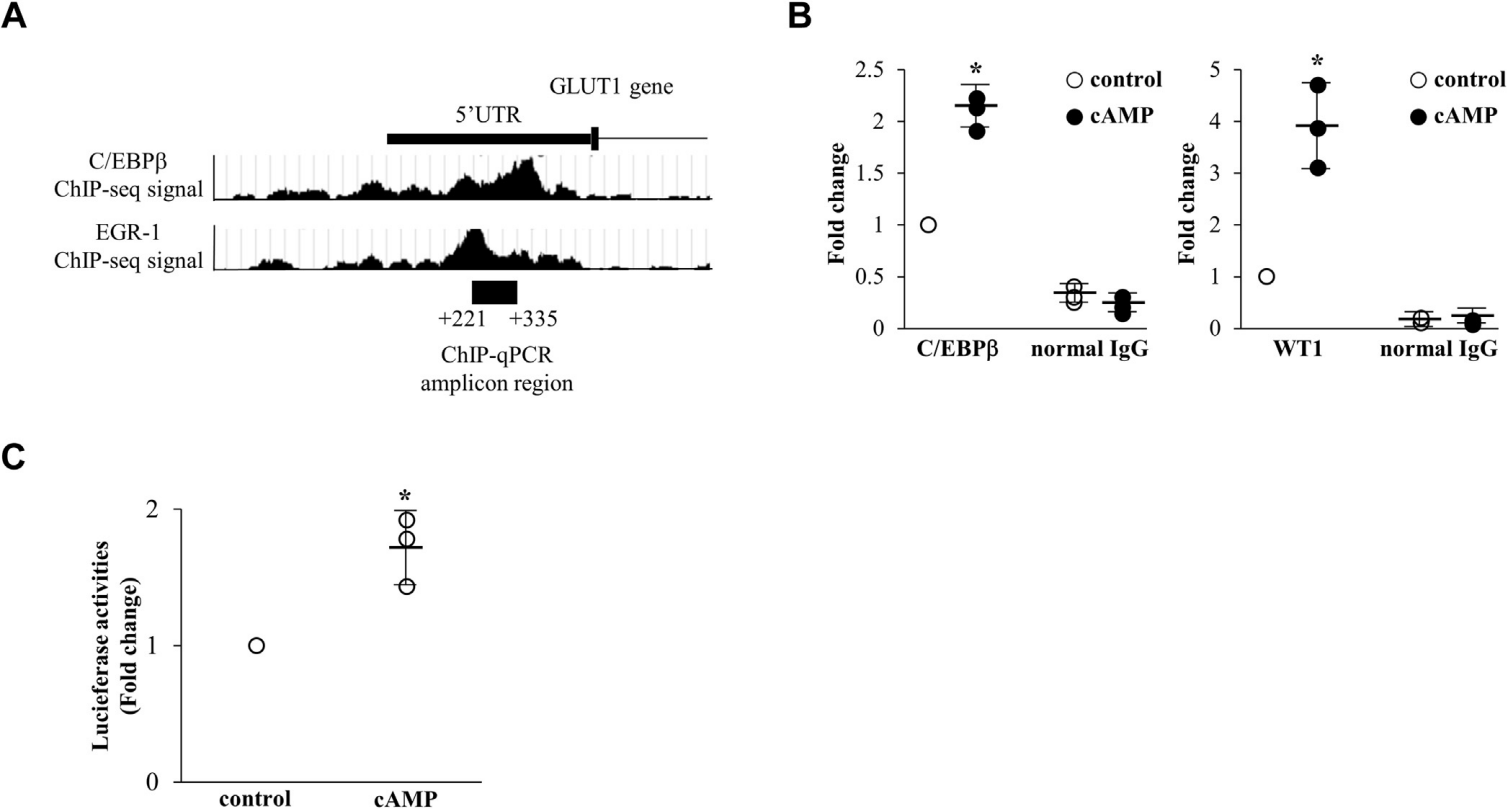
**Decidualization-induced recruitment of C/EBPβ and WT1 to the GLUT1 promoter region.***A*, ChIP-sequence data on the binding of C/EBPβ and EGR-1 in HCT-116 cells from ENCODE. Because WT1 shares the same DNA consensus binding sequence with EGR-1, the binding signals of C/EBPβ and EGR-1 were observed in the 5′ UTR region of the GLUT1 gene. ChIP-qPCR was performed in ESCs with the primers surrounding the binding sequences of C/EBPβ and EGR-1 (+221 to +335 bp). The *black bar* at the *bottom* indicates the ChIP-qPCR amplicon region. *B*, effects of cAMP on the recruitment of C/EBPβ and WT1 to the GLUT1 promoter region. ESCs were treated with or without cAMP for 4 days. The recruitments of C/EBPβ and WT1 were examined by ChIP assay. Normal rabbit IgG was used as a negative control. The relative recruitment levels were analyzed by real-time PCR. Values were expressed as a ratio of the control sample. Data are mean ± SD of three different incubations. ∗*p* < 0.01 *versus* control treatment (unpaired *t* test). *C*, effect of cAMP on the transcriptional activity of the GLUT1 promoter region. ESCs were transfected with the GLUT1-promoter reporter vector and pRL-TK vector as a normalization control. After 24 h of transfection, cells were treated with or without cAMP for 4 days. The firefly luciferase activity was normalized to those of Renilla. Values of the luciferase activities were expressed as a ratio of the control sample. Data are mean ± SD of three different incubations. ∗*p* < 0.01 *versus* control treatment (unpaired *t* test).

To clarify the mechanism by which C/EBPβ and WT1 regulate GLUT1 mRNA expression during decidualization, we searched the promoter region of GLUT1 for potential C/EBPβ and WT1 binding sites. WT1 binds to the 9 bp DNA sequence GCG(G/T)GGGCG, which is similar to the consensus binding sequence recognized by early growth response-1 (EGR-1) (22, 23). Therefore, we searched ChIP-sequence data in the Encyclopedia of DNA Elements (ENCODE) project for binding signals of C/EBPβ and EGR-1 around the GLUT1 promoter region in HCT-116 cell (24), because it is a human colon cancer cell line that highly expresses GLUT1 (25). As shown in Figure 4*A*, the binding signals of both C/EBPβ and EGR-1 were observed in the 5′ UTR of the GLUT1 gene (Fig. 4*A*). The DNA sequences around the binding signals were submitted to the JASPAR database (http://jaspar.binf.ku.dk/), which predicted that the consensus binding sequences of C/EBPβ and WT1 were close together (Fig. S1). Then, ChIP-qPCR primers were designed to cover these predicted sequences to examine the recruitments of C/EBPβ and WT1 (+221 to +335 bp) (Fig. 4*A* and Fig. S1). ChIP-qPCR revealed that cAMP significantly increased the recruitment of both C/EBPβ and WT1 to the GLUT1 promoter region (Fig. 4*B*). To determine whether the promoter region has transcriptional activities, a luciferase construct containing the GLUT1 promoter region was transfected into ESCs. cAMP increased luciferase activities of the promoter regions (Fig. 4*C*). These results showed that C/EBPβ and WT1 upregulate GLUT1 expression by binding to the GLUT1 promoter region during decidualization.

### Involvement of H3K27ac in the regulation of GLUT1 expression during decidualization

**Figure 5:**
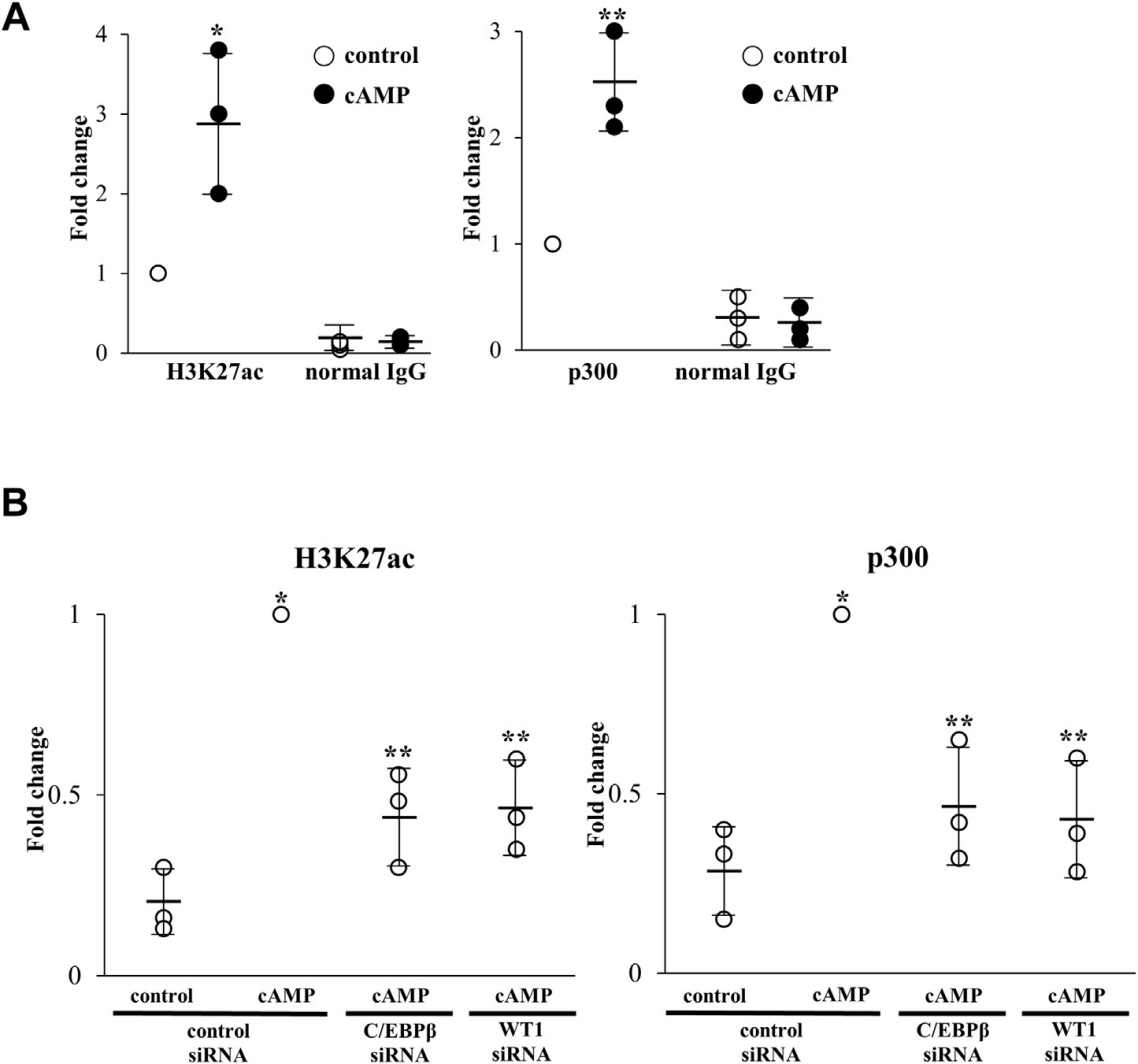
**Involvement of H3K27ac in the regulation of GLUT1 expression during decidualization.***A*, effects of cAMP on the levels of H3K27ac (*left*) and p300 recruitment (*right*) in the GLUT1 promoter region. ESCs were treated with or without cAMP for 4 days. The levels of H3K27ac and p300 recruitment were examined by ChIP assay. Normal mouse (for H3K27ac) or rabbit (for p300) IgG was used as a negative control. The relative levels were analyzed by real-time PCR. Values were expressed as a ratio of the control sample. Data are mean ± SD of three different incubations. ∗*p* < 0.01 *versus* control treatment; ∗∗*p* < 0.05 *versus* control treatment (unpaired *t* test). *B*, effects of knockdown of C/EBPβ or WT1 on the levels of H3K27ac (*left*) and p300 recruitment (*right*) in the GLUT1 promoter region. Forty-eight hours after siRNA transfection against C/EBPβ or WT1, ESCs were treated with or without cAMP for 4 days. The levels of H3K27ac and p300 recruitment were examined by ChIP assay. The relative levels were analyzed by real-time PCR. Values were expressed as a ratio of the cAMP treatment sample in the control siRNA sample. Data are mean ± SD of three different incubations. ∗*p* < 0.01 *versus* control treatment in the control siRNA; ∗∗*p* < 0.05 *versus* cAMP treatment in the control siRNA (Tukey–Kramer test).

H3K27ac is a histone modification that activates gene transcription (19). Our previous ChIP-sequence analysis revealed that the H3K27ac levels at many genomic loci increase during decidualization (4). The GLUT1 promoter was one of these regions, suggesting that H3K27ac is involved in the upregulation of GLUT1 during decidualization. First, we validated the results of the ChIP-sequence analysis by ChIP-qPCR, which confirmed that cAMP treatment significantly increases the H3K27ac level of the GLUT1 promoter region (Fig. 5*A*). p300 is a transcription coactivator that has histone acetyltransferase (HAT) activities and induces H3K27ac (26, 27, 28). p300 interacts with both C/EBPβ and WT1 (29, 30, 31). Therefore, we examined the effect of cAMP on the p300 recruitment to the GLUT1 promoter region. cAMP significantly increased the recruitment of p300 (Fig. 5*A*), which suggests that this is the mechanism by which cAMP increases H3K27ac levels. In general, chromatin remodeling regulated by histone modifications is induced by the recruitment of pioneer transcription factors (32, 33), which recruit cofactors with HAT activities. Because p300 colocalized on the binding sites of C/EBPβ and WT1 in the GLUT1 promoter region, we hypothesized that C/EBPβ and WT1 are involved in the induction of H3K27ac by recruiting p300. To test this hypothesis, the effects of knockdown of C/EBPβ or WT1 on the levels of H3K27ac and p300 recruitment were examined. Knockdown of either C/EBPβ or WT1 significantly inhibited the cAMP-increased H3K27ac levels and p300 recruitment (Fig. 5*B*), indicating that they both contribute to the increase of H3K27ac by recruiting p300 to the GLUT1 promoter region during decidualization.

## Discussion

Drastic changes of cellular metabolisms occur in ESCs during decidualization. One of these changes is glucose metabolism. We previously reported that glucose uptake increases during decidualization and that glucose is indispensable for decidualization (5, 8). However, it is not clear how glucose uptake is regulated in ESCs. The present study showed that GLUT1 is involved in the increase of glucose uptake of human ESCs during decidualization. Furthermore, we showed that GLUT1 is regulated by C/EBPβ and WT1 in an epigenetic manner.

GLUT 1 is responsible for the basal uptake and storage of glucose rather than insulin-dependent glucose uptake (34) and is especially abundant in erythrocytes and the hemochorial placenta (9). It has been reported that GLUT1 is highly expressed in the human endometrium, whereas the expressions of other GLUTs, including insulin-dependent glucose uptake (GLUT2, GLUT4, and GLUT8), were very low or not detected (10, 11), which is supported by our transcriptome data. Previous studies have shown that GLUT1 is upregulated during decidualization and is involved in the induction of IGFBP-1 and PRL expression (10, 11). However, none of these studies examined the direct effect of GLUT1 knockdown on the glucose uptake. Therefore, it has been unclear whether GLUT1 is involved in the glucose uptake in ESCs. In this study, we clearly demonstrated that GLUT1 knockdown suppressed the increase of glucose uptake by decidualization with the suppression of IGFBP-1 and PRL expressions. Considering that glucose is essential for decidualization (5, 8), GLUT1 contributes to decidualization by regulating glucose uptake in human ESCs. In fact, reduced stromal expression of GLUT1 has been observed in infertile women (10). Taken together, these findings indicate that the upregulation of GLUT1 is an essential event for decidualization and the subsequent establishment of pregnancy.

Regarding the glucose metabolic pathways activated during decidualization, it was reported that decidualization stimulus activates the glycolysis pathway (35). In addition, according to our gene ontology analysis of our transcriptome data (4), the terms related to the glycolysis pathway were enriched in the cAMP-upregulated genes. Therefore, we speculate that the increased glucose uptake by cAMP is associated with the activation of glycolysis pathway. Although the role of the activated glucose in decidualization remains unknown, it would be interesting what kinds of glucose metabolic pathways are activated and how they are involved in decidualization.

Although the PI3K/AKT and MAPK pathways were recently shown to be involved (36), the transcriptional regulation of GLUT1 in ESCs remains unclear. The present study showed that C/EBPβ and WT1 upregulate GLUT1 expression through the binding to the promoter region. Although several transcription factors regulating GLUT1 have been identified in cells other than ESCs (37, 38), this is the first report showing the involvement of C/EBPβ and WT1 in the regulation of GLUT1 in ESCs as well as any other types of cells. C/EBPβ is a critical transcription factor for decidualization because C/EBPβ knockout impairs decidualization in mice (39). We previously reported that C/EBPβ regulates many decidualization-related gene expressions in human ESCs, which include genes involved in cellular metabolisms (4). WT1 is a well-known transcription factor for the regulation of mammalian urogenital development (40). We previously showed that WT1 is involved in the regulation of decidualization (15). Furthermore, our recent report showed that WT1 regulates lipid uptake through the increase of very low-density lipoprotein receptor (VLDLR) expression during decidualization (6). So far, there have been no reports showing that C/EBPβ or WT1 is involved in the regulation of glucose uptake. Therefore, the present study demonstrates a novel function of C/EBPβ and WT1, in which they regulate glucose uptake through the increase of GLUT1. However, neither direct GLUT1 knockdown or indirect GLUT1 knockdown by C/EBPβ- or WT1-siRNA completely suppressed cAMP-increased expressions of IGFBP-1 and PRL. These results indicated that other factors than GLUT1-dependent glucose uptake are also playing a role in the regulation of decidualization. Therefore, our study shows one of the mechanisms for the regulation of decidualization.

The present results also demonstrate the epigenetic regulation of GLUT1. H3K27ac is a histone modification that activates gene transcription and is highly enriched in the active promoter or enhancer regions (19, 20). Our previous reports suggested a close relationship between H3K27ac and glucose metabolism during decidualization (5, 8). In addition, our recent ChIP-sequence analysis indicated that the H3K27ac level of the GLUT1 promoter region increases during decidualization (4), which led us to investigate the involvement of epigenetic regulation in GLUT1 expression. The present study demonstrated that upregulation of GLUT1 is accompanied by the increase of H3K27ac at the promoter region. Furthermore, we revealed the mechanisms by which cAMP increases H3K27ac levels of the GLUT1 promoter region. In general, chromatin remodeling regulated by histone modifications is induced by the recruitment of pioneer transcription factors, which is responsible for the initiation of the epigenetic changes (32, 33, 41). These factors recruit cofactors that have histone acetyltransferase (HAT) activities, such as p300, to induce H3K27ac (27, 28). C/EBPβ has been suggested to act as a pioneer factor in a variety of cells (41, 42, 43, 44, 45). We recently found that C/EBPβ contributes to the induction of H3K27ac by recruiting p300 at many promoter and enhancer regions during decidualization (4, 14), which included the GLUT1 promoter region. In this study, we showed that C/EBPβ knockdown suppressed the cAMP-increased p300 recruitment and H3K27ac levels in the GLUT1 promoter region, indicating that C/EBPβ contributes to the induction of H3K27ac by recruiting p300. It is interesting to note that WT1 knockdown also showed the same effects as those observed by C/EBPβ knockdown, indicating that WT1 contributes to the increase of H3K27ac in the GLUT1 promoter region by recruiting p300 as well as C/EBPβ. This is supported by a previous report showing that WT1 works as a pioneer factor by recruiting p300 and induces chromatin remodeling during kidney development (31). It was reported that multiple pioneer factors can simultaneously bind to *cis*-elements and recruit HAT factors cooperatively when their DNA binding sites are close together (46, 47). In fact, DNA-binding sites of C/EBPβ and WT1 in the GLUT1 promoter region are located close to each other (Fig. S1). This strongly suggests that they both contribute to the increase of H3K27ac by cooperatively recruiting p300 in the GLUT1 promoter region.

The present study is the first to show that GLUT1 is involved in the increase of glucose uptake in ESCs during decidualization. The upregulation of GLUT1 is mediated by the promoter bindings of C/EBPβ and WT1. Furthermore, both C/EBPβ and WT1 contribute to induce the epigenetic changes of the GLUT1 promoter by recruiting p300. Given that glucose is essential for decidualization, epigenetic regulation of GLUT1 is an important mechanism for the induction of decidualization.

## Experimental procedures

### Reagents

DMEM, L-glutamine, 1× trypsin–EDTA, streptomycin, and penicillin were purchased from Invitrogen. Fetal bovine serum (FBS) was obtained from Biological Industries Ltd. Collagenases, dibutyryl-cAMP (db-cAMP) were obtained from Sigma Chemical Co Ltd. Tissue flasks were from Becton Dickinson Co Ltd.

### ESC isolation

Human endometrial tissues were obtained at hysterectomy from patients with a normal menstrual cycle, aged 40 to 45 years, who underwent surgery for myoma uteri or early-stage cervical cancer. The patients were not on hormonal therapy at the time of surgery. Informed consent was obtained from all participating patients, and ethical approval was obtained from the Institutional Review Board of Yamaguchi University Hospital. All experiments were performed in accordance with the Tenets of the Declaration of Helsinki. Endometrial samples utilized for ESC isolation were histologically diagnosed as being in the late proliferative phase according to published criteria (48). Tissue samples were washed with Phenol Red-free DMEM containing 4 mM glutamine, 50 μg/ml streptomycin, and 50 IU/ml penicillin and minced into pieces of <1 mm^3^. ESCs were isolated as reported previously (17). In brief, tissues were minced, enzymatically digested with 0.2% collagenase in a shaking water bath for 2 h at 37 °C, and filtered through a 70 μm nylon mesh. Stromal cells in the filtrates were washed three times with the medium, and the number of viable cells was counted by Trypan blue dye exclusion. Under the microscope, all of the cells reacted with the stromal-reacting antibody vimentin (data not shown), indicating that they were homogeneous. The cells were also verified to be negative for an epithelial cell-reacting antibody (cytokeratin) (data not shown). Cells were seeded at 10^5^ cells/cm^2^ in 75 cm^2^ tissue culture flasks and incubated in Phenol Red-free DMEM containing glutamine, antibiotics, and 10% dextran-coated charcoal-stripped FBS at 37 °C, 95% air, and 5% CO_2_. At confluence, cells were treated with 1× trypsin–EDTA and subcultured into 6-well plates. At 80% confluence after the first passage, the cell culture medium was changed to the treatment medium.

### Cell culture

To induce decidualization, ESCs were incubated with treatment medium (Phenol Red-free DMEM supplemented with glutamine, antibiotics, and 2% dextran-coated charcoal-stripped FBS) containing cAMP (0.5 mM) for 4 days. The cells were then used for the experiments described below. cAMP is considered as a second messenger of progesterone for decidualization because progesterone increases intracellular cAMP concentrations in ESCs (49). The concentration of cAMP (0.5 mM) and the period of incubation (4 days) used in this study were based on our previous report (49). The medium was changed every other day. A single incubation was performed in triplicate on cells isolated from an individual. We repeated the incubation with ESCs from three different individuals in each experimental procedure.

### Immunohistochemistry

Tissue samples from the late proliferative phase and late secretory phase were immunostained as reported previously (21). The endometrial tissues were fixed in formalin, embedded in paraffin, and cut into 5 μm sections. The sections were deparaffinized in xylene, dehydrated in a graded series of ethanol, stained with Histofine simple stain MAX-PO(R) (Nichirei Co Ltd) using a rabbit polyclonal antibody to GLUT1 (Abcam plc), incubated in 3,3′-diaminobenzidine-4 HCl (Nakalai Tesque Co) in 0.05 M Tris-HCl buffer (pH 7.6) containing 0.01% H_2_O_2_ for 3 min to visualize peroxidase activity and counterstained with Meyer’s hematoxylin. Control sections were incubated with normal rabbit IgG.

### Real-time RT-PCR

Total RNA was isolated from the cultured cells with an RNeasy Mini Kit. (Qiagen). The RNA was reverse transcribed as reported previously (50). For PCR amplification, first-strand cDNA was synthesized from 1 μg total RNA with reverse transcriptase (Invitrogen) in 20 μl of the reaction mixture. Amplicons of GLUT1, IGFBP-1, PRL, and MRPL19 were amplified by real-time RT-PCR as reported previously (51, 52) with sequence-specific primer sets (Table S1). Cells isolated from one patient were incubated in triplicate. Mean expression values of three wells were calculated in each treatment group, respectively. Then, the fold change of mean value in each group relative to the mean value in control group was determined. This is a result from an individual/an incubation. We repeated the same incubations with two more different individuals. Then, the mean ± SD of fold change from three different individuals was used as a relative expression value in each treatment group.

### Western blotting

Western blotting was performed as reported previously (53, 54). In brief, whole cell lysates were prepared using loading buffer reagents (Santa Cruz Biotechnology, Inc) without trypsin treatment. Equal amounts of total protein were electrophoresed on a 10% SDS-polyacrylamide gel. The proteins were transferred to polyvinylidene difluoride membranes (ATTO). The membranes were blocked with blocking solution [5% skimmed milk with 0.1% Tween-20 dissolved in Tris-buffered saline (pH 7.5)], incubated with the first antibody for GLUT1 (Abcam), C/EBPβ (Santa Cruz Biotechnology), WT1(Abcam), and β-tubulin (Sigma), which were diluted in blocking solution, incubated with the peroxidase-conjugated second antibody diluted in blocking solution, visualized with the ECL-Western blotting detection system (Amersham) according to the manufacturer's protocol, and used to expose hyperfilm-ECL (Amersham). To reuse the blot, the membranes were stripped in Restore Western stripping buffer (Pierce). Western blot bands were quantified by ImageJ. The uncropped images of immunoblots are shown in Fig. S2.

### Lipid-mediated transfection of small interfering RNA (siRNA) duplexes

GLUT1 ON-TARGET plus SMART pool, C/EBPβ ON-TARGET plus SMART pool, WT1 ON-TARGET plus SMART pool, and ON-TARGET plus Non-Targeting pool siRNA were purchased from Dharmacon. ESCs at 50% confluence were transfected with siRNA duplexes (20 nM) and RNAi MAX (Invitrogen) as we reported previously (6). The medium was changed 5 h later. After 48 h of transfection, cells were incubated with or without cAMP for 4 days and then used for each experiment.

### Glucose uptake assay

The levels of glucose uptake in ESCs were determined using a 2-deoxyglucose (DG) uptake assay kit (Cosmo-bio) as reported previously (5). ESCs were incubated with serum and glucose-free DMEM for 6 h. The cells were washed three times with Krebs-Ringer-phosphate-Hepes (KRPH) buffer (20 mM Hepes, 5 mM KH_2_PO_4_, 1 mM MgSO_4_, 1 mM CaCl_2_, 136 mM NaCl and 4.7 mM KCl, pH 7.4, 37 °C) containing 2% bovine serum albumin (BSA) and were further incubated at 37 °C for 20 min with KRPH buffer containing 2% BSA and 0.1 mM 2-DG. Then, the cells were washed three times with PBS, collected in 400 μl of 10 mM Tris-HCl (pH 8.0), heated at 80 °C for 15 min, and centrifuged at 15,000*g* for 20 min at 4 °C. The supernatants were assayed for a 2-DG uptake measurement. The total protein concentration of the cultured cells was used for normalization.

### ChIP assay

The levels of transcription factor recruitment and H3K27ac levels were examined by ChIP assay according to the protocol for the ChIP assay kit (Upstate Biotechnology) as we reported previously (6, 14). Cells were cross-linked by the addition of formaldehyde into the medium at a final concentration of 1% and incubated for 10 min at 37 °C. Cross-linking was terminated by the addition of glycine (0.125 M, final concentration). Cells lysates were sonicated using a Bioruptor ultrasonicator (Cosmo-bio), precleared with salmon sperm DNA-protein A at 4 °C for 4 h. Five percent of the supernatants were kept as input controls (INPUT). Dynabeads Protein A (Invitrogen) were incubated with antibodies for WT1 (Santa Cruz Biotechnology), C/EBPβ (Santa Cruz Biotechnology), p300 (Abcam), H3K27ac (generous gift from Dr H. Kimura) (55), and normal mouse or rabbit IgG (Invitrogen) 4 °C overnight. The precleared chromatin was incubated with antibody-bound Dynabeads for 8 h at 4 °C to collect the immune complexes. The cross-linking of the immunoprecipitated chromatin complex (IP) and input control (INPUT; 2% of the total soluble chromatin) were reversed by heating the samples at 65 °C overnight and then subjected to proteinase K treatment. The DNA was purified using a QIAquick PCR purification kit (QIAGEN) and used as a template for PCR amplification with various primer sets (Table S1) to the GLUT1 promoter region. Real-time PCR was used to determine the relative levels of C/EBPβ, WT1, and p300 recruitment and H3K27ac levels of the GLUT1 promoter region. The ratio of IP DNA to the INPUT DNA sample (%INPUT) was calculated as reported previously (14).

### Luciferase assay

The GLUT1 promoter region was amplified with the same primer sequences used for ChIP assay. The PCR product was subcloned upstream of the luciferase gene within firefly luciferase vectors pGL4.23 (Promega), which contains minimal promoter. ESCs were cultured on a 24-well plate (5 × 10^4^ cells/well) for 24 h and then transfected with a reporter vector and pRL-TK vector (Promega) as a normalization control using Lipofectamine LTX (Invitrogen). After 24 h of transfection, cells were treated in the presence or absence of cAMP for 4 days. The firefly and Renilla luciferase activities were measured using a Dual-Luciferase Reporter Assay System (Promega) according to the manufacturer’s instructions.

### Statistical analysis

Statistical significance was determined by one-way ANOVA. After ANOVA, the Tukey–Kramer test was applied to analyze differences between groups. An unpaired *t* test was applied to analyze the difference between the two groups. All statistical analyses were performed using SPSS for Windows version 11 (SPSS Inc). Differences were considered significant at *p* < 0.05.

## Data availability

All data are contained within the manuscript.

## Supporting information

This article contains supporting information.

## Conflict of interest

The authors declare that they have no conflicts of interest with the contents of this article.
