# Supplementary material for: The essential glucose transporter GLUT1 is epigenetically upregulated by C/EBPβ and WT1 during decidualization of the endometrium
Source: J Biol Chem. 2021 Aug 31;297(4):101150. doi: 10.1016/j.jbc.2021.101150 (PMC8458984; doi:10.1016/j.jbc.2021.101150)
Supplement: Supplemental Figures S1, S2 and Table S1 [file mmc1.docx]

Supplemental material to

**The essential glucose transporter GLUT1 is epigenetically upregulated by C/EBPβ and WT1 during decidualization of the endometrium**

Isao Tamura*, Taishi Fujimura, Yumiko Doi-Tanaka, Haruka Takagi, Yuichiro Shirafuta, Takuya Kajimura, Yumiko Mihara, Ryo Maekawa, Toshiaki Taketani, Shun Sato, Hiroshi Tamura, Norihiro Sugino

Including

Table S1

Figure S1-S2

**Table S1. Primer sequences used in this study**

| ChIP-qPCR | Forward | Reverse |
| --- | --- | --- |
| GLUT1 promoter (for transcription factor recruitment) | GAGGTCCTGCCCACACAC | GCTCGCTGTTGCTACCTCTT |
| GLUT1 promoter (for H3K27ac) | AACGAAAACAGCCTCACTGG | CTGGGACGCCTTCCTCTACT |
|  |  |  |
|  |  |  |
| Real-time RT-PCR | Forward | Reverse |
| GLUT1 | CTTCACTGTCGTGTCGCTGT | CCAGGACCCACTTCAAAGAA |
| IGFBP-1 | CGAAGGCTCTCCATGTCACCA | TGTCTCCTGTGCCTTGGCTAAAC |
| PRL | AAAGGATCGCCATGGAAAG | GCACAGGAGCAGGTTTGAC |
| MRPL19 | GAATGTTATCGAAGGACAAGGT | CAGGAAGGGCATCTCGTAAG |


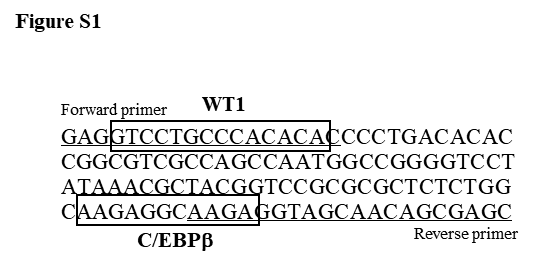


**Figure S1. DNA sequences of the GLUT1 promoter region.**  The consensus binding sequences of C/EBPβ and WT1 are boxed. Primer sequences for ChIP-qPCR are underlined.


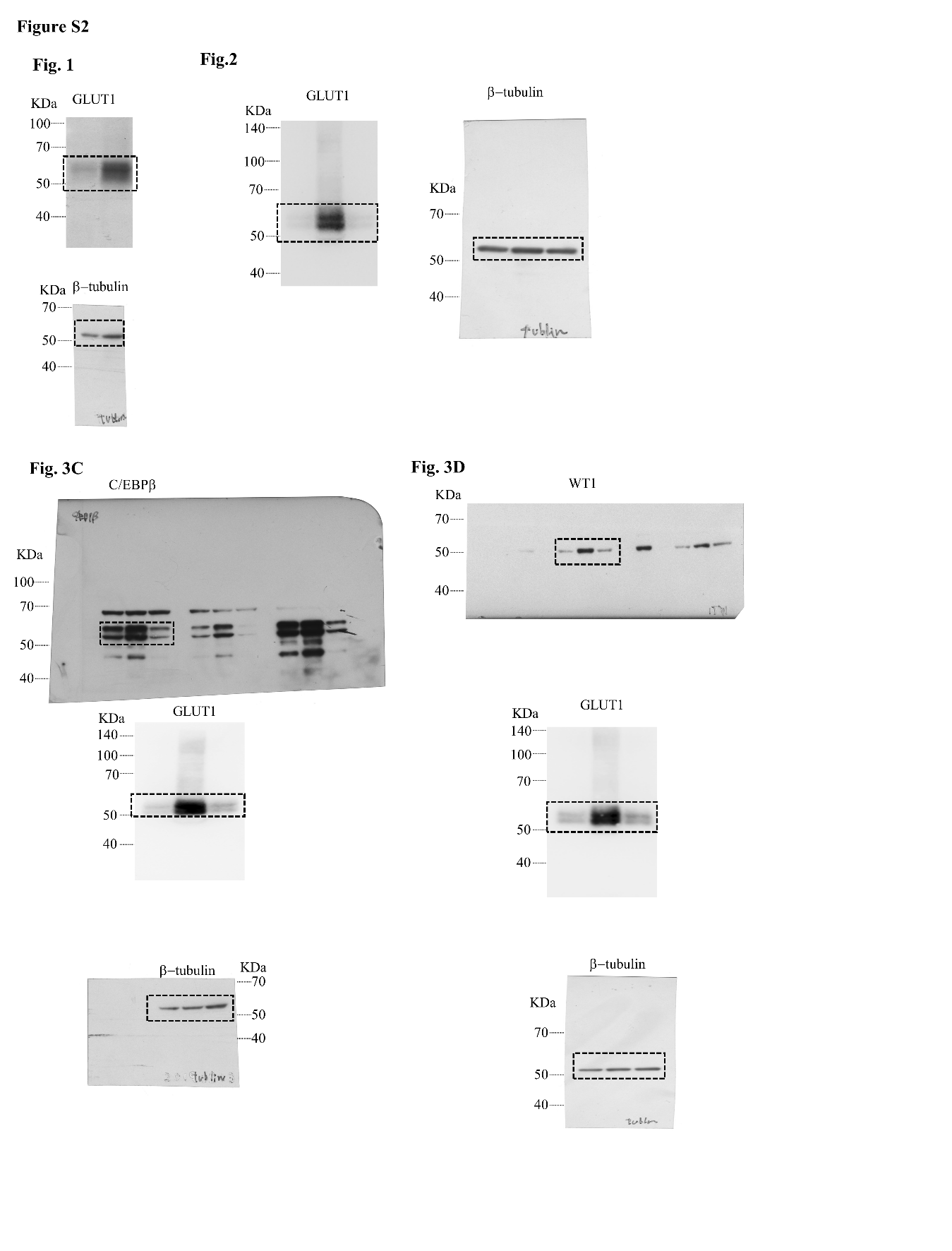


**Figure S2. Uncropped images of the immunoblots of each figure.**  The cropped areas in each figure are indicated by dotted lines.
